# Supplementary material for: HIVprotI: an integrated web based platform for prediction and design of HIV proteins inhibitors
Source: J Cheminform. 2018 Mar 9;10:12. doi: 10.1186/s13321-018-0266-y (PMC5845081; doi:10.1186/s13321-018-0266-y)
Supplement: Supplementary file 2 — Additional file 2. Source code of HIVProtI web server. [file 13321_2018_266_MOESM2_ESM.zip › HIVprotI_Source-code/hivprotI/weka-3-6-6/doc/weka/core/CapabilitiesHandler.html]

CapabilitiesHandler


---


|  |  |  |
| --- | --- | --- |
| **Overview** \| **Package** \| **Class** \| **Tree** \| **Deprecated** \| **Index** \| **Help** \| **Weka's home** | |  |
| **PREV CLASS**   **NEXT CLASS** | **FRAMES**    **NO FRAMES**     **All Classes** |  |
| SUMMARY: NESTED \| FIELD \| CONSTR \| METHOD | DETAIL: FIELD \| CONSTR \| METHOD |  |


---


## weka.core Interface CapabilitiesHandler

**All Known Subinterfaces:**: MultiInstanceCapabilitiesHandler

**All Known Implementing Classes:**: AbstractAssociator, AbstractClusterer, AbstractDensityBasedClusterer, AbstractFileSaver, AbstractSaver, AbstractTimeSeries, AdaBoostM1, Add, AddClassification, AddCluster, AddExpression, AddID, AdditiveRegression, AddNoise, AddValues, ADTree, AllFilter, AODE, AODEsr, Apriori, ArffSaver, ASEvaluation, AttributeSelectedClassifier, AttributeSelection, AttributeSetEvaluator, Bagging, BayesianLogisticRegression, BayesNet, BayesNetGenerator, BFTree, BIFReader, C45PruneableClassifierTree, C45PruneableClassifierTreeG, C45Saver, CachedKernel, Center, CfsSubsetEval, ChangeDateFormat, ChiSquaredAttributeEval, CitationKNN, ClassAssigner, ClassBalancedND, ClassificationViaClustering, ClassificationViaRegression, Classifier, ClassifierSubsetEval, ClassifierTree, ClassOrder, CLOPE, ClusterMembership, Cobweb, ComplementNaiveBayes, ConjunctiveRule, ConsistencySubsetEval, Copy, CostSensitiveASEvaluation, CostSensitiveAttributeEval, CostSensitiveClassifier, CostSensitiveSubsetEval, CSVSaver, CVParameterSelection, Dagging, DatabaseSaver, DataNearBalancedND, DBScan, DecisionStump, DecisionTable, Decorate, DiscreteEstimator, DiscreteEstimatorBayes, DiscreteEstimatorFullBayes, Discretize, Discretize, DMNBtext, DTNB, EditableBayesNet, EM, END, Estimator, FarthestFirst, Filter, FilteredAssociator, FilteredAttributeEval, FilteredClassifier, FilteredClusterer, FilteredSubsetEval, FindWithCapabilities, FirstOrder, FPGrowth, FT, FTInnerNode, FTLeavesNode, FTNode, FTtree, GainRatioAttributeEval, GaussianProcesses, GeneralizedSequentialPatterns, GeneralRegression, Grading, GridSearch, HierarchicalClusterer, HNB, HoldOutSubsetEvaluator, HyperPipes, IB1, IBk, Id3, InfoGainAttributeEval, InterquartileRange, IsotonicRegression, IteratedSingleClassifierEnhancer, J48, J48graft, JRip, Kernel, KernelEstimator, KernelFilter, KStar, LADTree, LatentSemanticAnalysis, LBR, LeastMedSq, LibLINEAR, LibSVM, LibSVMSaver, LinearRegression, LMT, LMTNode, Logistic, LogisticBase, LogitBoost, LWL, M5Base, M5P, M5Rules, MahalanobisEstimator, MakeDecList, MakeDensityBasedClusterer, MakeIndicator, MathExpression, MDD, MergeTwoValues, MetaCost, MIBoost, MIDD, MIEMDD, MILR, MINND, MIOptimalBall, MIPolyKernel, MIRBFKernel, MISMO, MISVM, MIWrapper, MultiBoostAB, MultiClassClassifier, MultiFilter, MultiInstanceToPropositional, MultilayerPerceptron, MultipleClassifiersCombiner, MultiScheme, NaiveBayes, NaiveBayesMultinomial, NaiveBayesMultinomialUpdateable, NaiveBayesSimple, NaiveBayesUpdateable, NBTree, NBTreeClassifierTree, ND, NeuralNetwork, NNge, NominalToBinary, NominalToBinary, NominalToString, NonSparseToSparse, NormalEstimator, Normalize, Normalize, NormalizedPolyKernel, NumericCleaner, NumericToBinary, NumericToNominal, NumericTransform, Obfuscate, OneR, OneRAttributeEval, OPTICS, OrdinalClassClassifier, PaceRegression, PART, PartitionedMultiFilter, PKIDiscretize, PLSClassifier, PLSFilter, PMMLClassifier, PoissonEstimator, PolyKernel, PotentialClassIgnorer, PrecomputedKernelMatrixKernel, PreConstructedLinearModel, PredictiveApriori, PrincipalComponents, PrincipalComponents, Prism, PropositionalToMultiInstance, PruneableClassifierTree, Puk, RacedIncrementalLogitBoost, RandomCommittee, RandomForest, RandomizableClassifier, RandomizableClusterer, RandomizableDensityBasedClusterer, RandomizableIteratedSingleClassifierEnhancer, RandomizableMultipleClassifiersCombiner, RandomizableSingleClassifierEnhancer, RandomizableSingleClustererEnhancer, Randomize, RandomProjection, RandomSubset, RandomSubSpace, RandomTree, RBFKernel, RBFNetwork, Regression, RegressionByDiscretization, RELAGGS, ReliefFAttributeEval, Remove, RemoveFolds, RemoveFrequentValues, RemoveMisclassified, RemovePercentage, RemoveRange, RemoveType, RemoveUseless, RemoveWithValues, Reorder, ReplaceMissingValues, REPTree, Resample, Resample, ReservoirSample, Ridor, RotationForest, RuleNode, SerializedClassifier, SerializedInstancesSaver, sIB, SimpleBatchFilter, SimpleCart, SimpleFilter, SimpleKMeans, SimpleLinearRegression, SimpleLogistic, SimpleMI, SimpleStreamFilter, SingleAssociatorEnhancer, SingleClassifierEnhancer, SingleClustererEnhancer, SMO, SMOreg, SMOTE, SparseToNonSparse, SPegasos, SpreadSubsample, Stacking, StackingC, Standardize, StratifiedRemoveFolds, StringKernel, StringToNominal, StringToWordVector, SubsetByExpression, SVMAttributeEval, SVMLightSaver, SwapValues, SymmetricalUncertAttributeEval, Tertius, ThresholdSelector, TimeSeriesDelta, TimeSeriesTranslate, UnsupervisedAttributeEvaluator, UnsupervisedSubsetEvaluator, UserClassifier, VFI, Vote, VotedPerceptron, WAODE, Wavelet, Winnow, WrapperSubsetEval, XMeans, XRFFSaver, ZeroR

---

``` public interface CapabilitiesHandler ```

Classes implementing this interface return their capabilities in regards
to datasets.

**Version:**
:   $Revision: 1.1 $

**Author:**
:   FracPete (fracpete at waikato dot ac dot nz)

**See Also:**: `Capabilities`

---

| **Method Summary** | |
| --- | --- |
| `Capabilities` | `getCapabilities()`             Returns the capabilities of this object. |

**Method Detail**

### getCapabilities

```
Capabilities getCapabilities()
```

:   Returns the capabilities of this object.

    :   **Returns:**: the capabilities of this object **See Also:**: `Capabilities`


---


|  |  |  |
| --- | --- | --- |
| **Overview** \| **Package** \| **Class** \| **Tree** \| **Deprecated** \| **Index** \| **Help** \| **Weka's home** | |  |
| **PREV CLASS**   **NEXT CLASS** | **FRAMES**    **NO FRAMES**     **All Classes** |  |
| SUMMARY: NESTED \| FIELD \| CONSTR \| METHOD | DETAIL: FIELD \| CONSTR \| METHOD |  |


---
